# Meeting Vitamin D Requirements in White Caucasians at UK Latitudes: Providing a Choice

**DOI:** 10.3390/nu10040497

**Published:** 2018-04-17

**Authors:** Ann R. Webb, Andreas Kazantzidis, Richard C. Kift, Mark D. Farrar, Jack Wilkinson, Lesley E. Rhodes

**Affiliations:** 1 School of Earth and Environmental Science, Faculty of Science and Engineering, University of Manchester, Manchester M13 9PL, UK; akaza@upatras.gr (A.K.); richard.kift@manchester.ac.uk (R.C.K.); 2 Physics Department, University of Patras, 265 00 Patras, Greece; 3 Faculty of Biology, Medicine and Health, University of Manchester, and Dermatology Centre, Salford Royal NHS Foundation Trust, Manchester Academic Health Science Centre, Manchester M6 8HD, UK; mark.farrar@manchester.ac.uk (M.D.F.); lesley.e.rhodes@manchester.ac.uk (L.E.R.); 4 Centre for Biostatistics, School of Health Sciences, Faculty of Biology, Medicine and Health, Manchester Academic Health Science Centre (MAHSC), University of Manchester, Manchester M13 9PL, UK; jack.wilkinson@manchester.ac.uk

**Keywords:** vitamin D, ultraviolet radiation, climatology, white Caucasian, dietary intake

## Abstract

The body gains vitamin D through both oral intake (diet/supplementation) and synthesis in skin upon exposure to ultraviolet radiation (UVR). Sun exposure is the major source for most people even though sun exposure is complex and limited by climate and culture. We aimed to quantify the sun exposure required to meet vitamin D targets year-round and determine whether this can be safely achieved in a simply defined manner in the UK as an alternative to increasing vitamin D oral intake. Data from observation (sun exposure, diet, and vitamin D status) and UVR intervention studies performed with white Caucasian adults were combined with modeled all-weather UVR climatology. Daily vitamin D effective UVR doses (all-weather) were calculated across the UK based on ten-year climatology for pre-defined lunchtime exposure regimes. Calculations then determined the time necessary to spend outdoors for the body to gain sufficient vitamin D levels for year-round needs without being sunburnt under differing exposure scenarios. Results show that, in specified conditions, white Caucasians across the UK need nine minutes of daily sunlight at lunchtime from March to September for 25(OH)D levels to remain ≥25 nmol/L throughout the winter. This assumes forearms and lower legs are exposed June-August, while in the remaining, cooler months only hands and face need be exposed. Exposing only the hands and face throughout the summer does not meet requirements.

## 1. Introduction

It is well established that vitamin D is essential for bone health, which promotes dietary calcium absorption and bone mineralization [1]. Deficiency can lead to rickets in children and to osteomalacia in children and adults. Recently, it has been reported that even seasonal, as opposed to year-round deficiency, is associated with significantly lower bone mineral density in white adolescents in the UK [2]. Moreover, there is evidence, although not conclusive, that vitamin D may assist in protecting against fracture risk in the elderly [3,4]. A range of other health benefits (e.g., immunomodulation, chemoprevention) have also been associated with vitamin D [5]. This has led to re-evaluation of vitamin D status and its sources. Vitamin D is naturally present in few foods (mainly in fatty fish and in smaller amounts in eggs and meat) and there is little vitamin D food fortification in the UK where dietary intake is low [6]. The majority of vitamin D is, therefore, acquired through skin synthesis, which is assumed by the national dietary guidance [7,8].

Vitamin D is formed in skin after exposure to ultraviolet-B radiation (UVB), which enters the bloodstream and undergoes hydroxylation in the liver to 25-hydroxyvitamin D (25(OH)D). This is the major circulating form of vitamin D and is used as a measure of vitamin D status. This is further hydroxylated in the kidney to 1,25-dihydroxyvitamin D (1,25(OH)_2_D), which is the active form [9]. In the UK, and at similar latitudes, summer midday sunlight contains enough UVB for vitamin D synthesis while the weaker sunlight of winter provides a negligible amount of vitamin D synthesis [10]. Therefore, vitamin D status declines throughout the winter [11]. To remain sufficient year-round, a relatively high circulating level of 25(OH)D must be achieved by the end of the summer through sun exposure. However, the cross-sectional National Diet and Nutrition Survey reveals that 8.4% of UK white 19–64 years old people have vitamin D deficiency (circulating 25(OH)D < 25 nmol/L) in the summertime, which rises to 39.3% in the winter [12] with a latitudinal pattern, i.e., higher 25(OH)D levels in Southern England than in Northern Scotland [13]. Longitudinal studies from north, mid, and south UK locations report a seasonal pattern in 25(OH)D levels that mirrors UVB availability and show a prevalence of vitamin D deficiency rising with increasing latitude from 0% to 8.6% in the summer and from 9.5% to 40.6% in the winter [11,14].

The risks of exposure to UV radiation (UVR; primarily sunburn, skin cancer) have formed part of public health advice for many years with UVR being the principal external cause of most skin cancers [15]. With recent suggestions that vitamin D might have multiple health benefits and improved understanding of vitamin D requirements for bone health, an apparent contradiction has arisen around public health guidance on sun exposure. The background to nutritional guidance on vitamin D [16] shows the original goal was to avoid rickets and osteomalacia, which was predicated on a white-skinned population and lifestyle of previous decades. Recent reassessment of nutritional guidance [3] has retained bone health as the basis for determining vitamin D status. However, we must now consider potential benefits of higher 25(OH)D levels, a modern lifestyle with potentially less outdoor activity, and an aging and multiethnic population with different social norms for skin exposure. In considering whether national dietary reference values for vitamin D should be amended, a quantifiable estimate of sun exposure necessary to meet vitamin D requirements is needed. Only then can this benefit of sunlight exposure be properly assessed and set into context against the uncertainties of dietary intake linked to food fortification or cost and potential compliance issues associated with dietary supplements.

Therefore, our objectives were to determine the low, sub-sunburn levels of sunlight exposure and the end-summer serum 25(OH)D target that provide an adequate year-round vitamin D status, which is defined as 25(OH)D level ≥25 nmol/L year-round by the national authorities [3] in the UK.

## 2. Materials and Methods

### 2.1. Underlying Data and Rationale

The sun exposure target is defined as one required to maintain winter 25(OH)D level ≥25 nmol/L based on the UK’s Scientific Advisory Committee on Nutrition (SACN) assessment of oral requirements [3]. While the SACN report has chosen to address the whole population with one easy message that suggests a Recommended Nutrient Intake (RNI) of 400 IU/day for everyone above one year of age [3], the choice of regular sunlight exposure remains for many who may wish to consider this as an alternative to increased dietary intake and/or a daily supplement. The sunlight exposure required to maintain a target 25(OH)D level throughout the year has been calculated using datasets from previously published studies [11,17,18] conducted in Greater Manchester, UK (Table 1). These datasets make direct in vivo examination of relationships between both natural sunlight and simulated sunlight exposure with vitamin D status outcomes in working-age and mixed-sex population groups with direct UK relevance.

**Figure 1:**
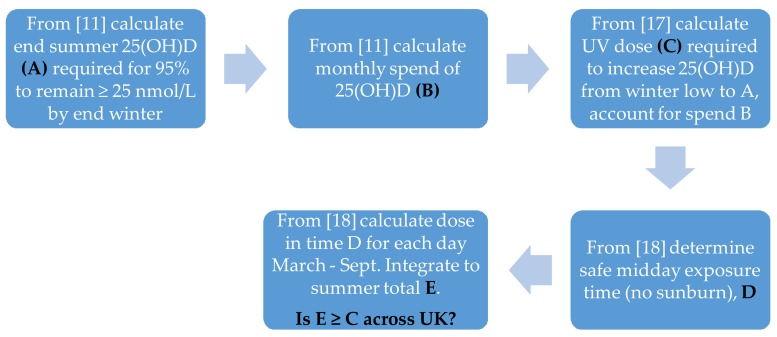
The sequence of analyses that led to the final sunlight exposure calculations for white Caucasians (skin types I–IV). Calculations do not consider any explicit dietary intake of vitamin D, but are based on results from volunteers who did acquire some oral vitamin D. The median was 3.26 (10–90 percentile, 0.91–7.81) µg/day (130.4 IU/day) [11]. Therefore, these small intakes are implicit in the end results.

Vitamin D status is measured by evaluating circulating 25(OH)D. Previous limits were aimed at avoiding deficiency (25(OH)D < 12.5–25 nmol/L) while 25(OH)D < 50 nmol/L is now classified insufficient in North America and several European countries [19]. In re-assessing vitamin D guidance, SACN defined the target vitamin D requirements for these calculations to be similar to nutritional requirements (i.e., 97.5% population remaining above deficiency: 25(OH)D ≥ 25 nmol/L). This was the starting point for calculating required sunlight exposure in the summertime (see Figure 1).

### 2.2. Defining the End Summer Target Levels of 25(OH)D

The September 25(OH)D level required for 25(OH)D ≥ 25 nmol/L by winter’s end (February) was determined from previous studies in white Caucasian adults [11]. Summer target levels for circulating 25(OH)D were calculated using linear regression techniques. Monthly 25(OH)D measurements [11] were log transformed in order to satisfy the assumptions of linearity and homoscedasticity of residuals underlying the analysis. February (minimum) 25(OH)D levels were then regressed on September (maximum) 25(OH)D levels in order to estimate the relationship between summer and winter levels. From this, the September levels were calculated, which were expected to result in 25 nmol/L by winter’s end. Although this is the expectation (average), individuals would fall both above and below this threshold. Therefore, September levels that an individual should attain in order to grant at least 95% probability of exceeding 25 nmol/L in February were calculated by constructing expressions for 95% prediction intervals for the expected winter levels, which resulted in different summer levels. The expressions were solved for September levels and produced intervals with lower limits greater than 25 nmol/L in the winter. This resulted in an end of September target of 80.5 nmol/L (A in Figure 1) for which 97.5% of individuals would exceed 25 nmol/L in February. The monthly spend of 25(OH)D was calculated from the decline in 25(OH)D from October to February (months when no cutaneous synthesis of vitamin D is expected in the UK) using data from Reference [11]. The monthly spend (B in Figure 1) is 6.25 nmol/L.

### 2.3. Relating a Change in Circulating 25(OH)D to Sun Exposure

Having determined A and B in Figure 1 and found the summer vitamin D synthesis required in terms of nmol/L 25(OH)D, we translated this into sunlight exposure, specifically the dose of UVR weighted with the pre-vitamin D action spectrum [21] (action spectrum defines the effectiveness of each wavelength in producing the stated biological effect). This specification must include skin type and the area exposed where both influence the change in 25(OH)D resulting from exposure to a given dose of UVR. Such information comes from the winter intervention study in Table 1 [17], which is conducted with volunteers wearing modest shorts and T-shirt to expose ~35% of skin area (the reference skin area) and given sub-sunburn UVR doses (equivalent to 1.1 Standard Erythemal Dose (SED) in sunlight)) three times weekly for six weeks to simulate repeated summer-time exposures. The SED is the selected unit of UVR exposure (1 SED = 100 Jm-2 erythema-weighted UVR [22]) since this is independent of personal response to UVR. A light-skinned person who does not easily tan would show a very mild sunburn after 2–3 SED. Change in 25(OH)D from beginning to end of the intervention study and total dose of vitamin D-weighted UVR to produce the change provided the nmol/L 25(OH)D per UVR dose needed for calculating the total summer sun exposure required (C in Figure 1).

Given that the September target 25(OH)D is 80.5 nmol/L and the mean February circulating 25(OH)D is 49.2 nmol/L [11], the required increase in circulating 25(OH)D over the warm and sunny half-year (mid-March to mid-September) is 31.3 nmol/L. The body uses vitamin D continuously, which is assumed to be a constant rate year-round. The observed vitamin D spend from October to February is 6.25 nmol/L/month [11]. Therefore, the summertime six-month production of vitamin D (expressed as circulating 25(OH)D) becomes: Production = Required change + summer spend = 31·3 + (6 × 6.25) = 68.8 nmol/L.

White Caucasion adult volunteers exposed to a sunlight equivalent dose of 1.1 SED three times per week for six weeks showed a mean rise in circulating 25(OH)D of 26 nmol/L [17]. This study was conducted in January and February when there is insufficient UVB in sunlight to initiate any significant vitamin D synthesis in skin. However, the spend during the six weeks (1.5 months) must be taken into account. The total production was 26 + (1.5 × 6.25) = 35·4 nmol/L, which resulted from exposure in the radiation cabinet to a total of 18 × 1.1 = 19.8 SED. Therefore, the production rate of 25(OH)D becomes 1.8 nmol/L/SED.

As such, to gain 68·8 nmol/L circulating 25(OH)D, the required UVR exposure is 68.8/1.8 = 38 SED (to the nearest whole SED). If we assume the source is the sun, the skin area exposed to the sun is 35% in the underlying study [17] and the exposure regime is little and often (i.e., a short exposure on a daily basis). Large, infrequent doses are inefficient for vitamin D synthesis because of the complexities of skin photochemistry [9]. It may also increase the risk of erythema and further skin damage.

The condition of 35% skin area exposed allows direct reference back to experimental results [17]. It is the equivalent of wearing a modest T-shirt and shorts or a skirt. In modifying the skin area exposed for other scenarios, it is assumed that skin on these exposed sites of the body synthesize vitamin D at a similar rate. In alternative scenarios where less skin area is exposed in cooler months of the year, the exposure time is kept constant and, therefore, the dose on any unprotected skin remains constant but the achievable dose for changing circulating 25(OH)D (daily inputs to E in Figure 1) has been scaled (% area exposed/35%) to represent the reduced skin exposure. The all-summer target dose remains at 38 SED.

### 2.4. Ensuring Health Risks from UVR Exposure Are Minimised

If advocating regular, albeit modest, sun exposure, it is necessary to ensure any associated risk is minimized, i.e., that erythema (sunburn, a proxy for skin cancer risk) does not occur. No erythema was experienced during the intervention study [17] so the same doses adjusted to the solar spectrum were taken as a starting point [23]. A further minor reduction was made for safety and the acceptable exposure dose was determined as 1 SED. The challenge then was to determine whether the all-summer vitamin D UVR dose could be achieved without ever exceeding the daily 1 SED dose given the UK climatology [18].

With multiple possibilities for exposure patterns, the shortest, safest, and most easily defined exposure regime was chosen. The greatest benefit to risk is when the sun is high in the sky [24] since there is the highest ratio of ambient UVB (for vitamin D synthesis) to UVA (which contributes to health risk). Therefore, exposure was restricted to hours immediately around solar noon. This is also when the solar intensity is greatest (reducing the time required), approximately constant, and coincides with lunchtime. To provide a simple message that could be used for public dissemination, exposures were expressed in units of time. Note that exposure during other periods of the day (e.g., in the early morning/late afternoon, equivalent to before/after work) would be far less effective and efficient for vitamin D synthesis and the exposure times required would be much longer than calculated below because the sun is lower in the sky and, therefore, there is less UVB irradiance.

Time required to achieve 1 SED at noon was calculated across the UK for clear skies. The baseline exposure scenario (S1, determined as the simplest public health message) was defined as time (D in Figure 1) to achieve 1 SED on a horizontal surface in southern England at noon on a clear day at the summer solstice (the time and place when the sun is most intense). This was set to nine minutes. Then the vitamin D-effective UVR dose was calculated for the same fixed time period for the entire United Kingdom and for each day of the summer season (March–September) by using all-weather climatology [18]. In the winter months, it was assumed that no appreciable vitamin D is made. Baseline skin area exposure of ~35% (face, hands, forearms, and lower legs) was assumed.

### 2.5. Translating from the Irradiation Cabinet to Real Life and from the Vertical to the Horizontal

All calculations thus far have referred to solar radiation falling on a horizontal surface and, in the irradiation cabinet studies [17], volunteers lay horizontally and were irradiated from above and below at the same time, three times per week. This situation is different in the sunlight since, when lying horizontal to sunbathe, only one side is exposed at a time. Therefore, exposure equivalent to that gained in the irradiation cabinet must be achieved in six ventral or dorsal single-side exposures, i.e., if on six days, this is nearly daily. It is also likely that a person going outside for a short time will be walking around and, thereby, exposing all uncovered skin at the same time (as in the irradiation cabinet), but not to a horizontal surface. This issue has been previously addressed [23] by calculating the exposure of a randomly oriented vertical surface (a proxy for the upright human body) with respect to the exposure of a horizontal surface in the same conditions for a range of solar zenith angles. These data were used to enable a conversion from the UVR climatology (horizontal surface) to a random vertical surface (proxy for ambulatory human body).

The total summertime UVR dose that would be achieved by a person following baseline exposure regime (S1: 9 min in the noontime hour, every day regardless of weather, walking around with 35% skin area exposed) was then calculated in the following way. The noon-hour horizontal dose for a day and location was taken from the climatological model and a percentage of this was calculated according to the exposure time (9/60 for the exposure of 9 min). The average solar zenith angle for that hour at that location was then calculated and used with data from Reference [23] to convert the horizontal dose to a random vertical dose. The random vertical doses were combined every day from March to September at a given location to give the achievable UVR dose under this exposure scenario (E in Figure 1). The achievable dose could then be compared with the required UVR dose (38 SED) assessed as necessary to meet vitamin D requirements.

### 2.6. Calculating Whether the End-Summer Target Can Be Achieved with Daily Sun Exposure for a Range of Exposure Scenarios

It should be noted that, although doses were converted for a random vertical orientation, the exposure time was based on horizontal exposures and this was retained. Even upright, parts of the human body are approximately horizontal (shoulders, top of head, nose, and top of feet) and the goal was to minimize the possibility of sunburn or sun damage of any sort. It is also recognized that people may sit or lie down and that a simple public health message should account for all situations. The exposure time, therefore, errs on the side of caution with respect to risk while the achievable UVR calculation (which we then relate to the benefit of vitamin D synthesis) is slightly reduced by using the random vertical assumption. Calculations were repeated for each scenario in Table 2 and accounted for temperature influences (exposing only hands and face (10% skin surface area) in March–May and September), cultural/social limits (exposing only hands and face all summer), and the holiday season (exposure only in June–August, but with time adapted to local climatology such as longer exposures (for the same UVR dose) in Scotland than in Southern England), respectively. In altering skin area, vitamin D synthesis was assumed equally effective for all uncovered skin and the outcome was scaled by the area exposed.

Finally, the details of what constitutes sufficient sunlight exposure to meet SACN-defined vitamin D needs is described in a form suitable for a simple public health message.

While our numerical results are specific to the UK, the methods used can be applied to any location. We also illustrate the variation that occurs, climatologically, across the 10-degree latitude band that covers the British Isles, which allows for analogy with locations of similar latitudes.

Details of analytical methods are provided within the body of this work. The statistical software used throughout was R [25].

## 3. Results

**Figure 2:**
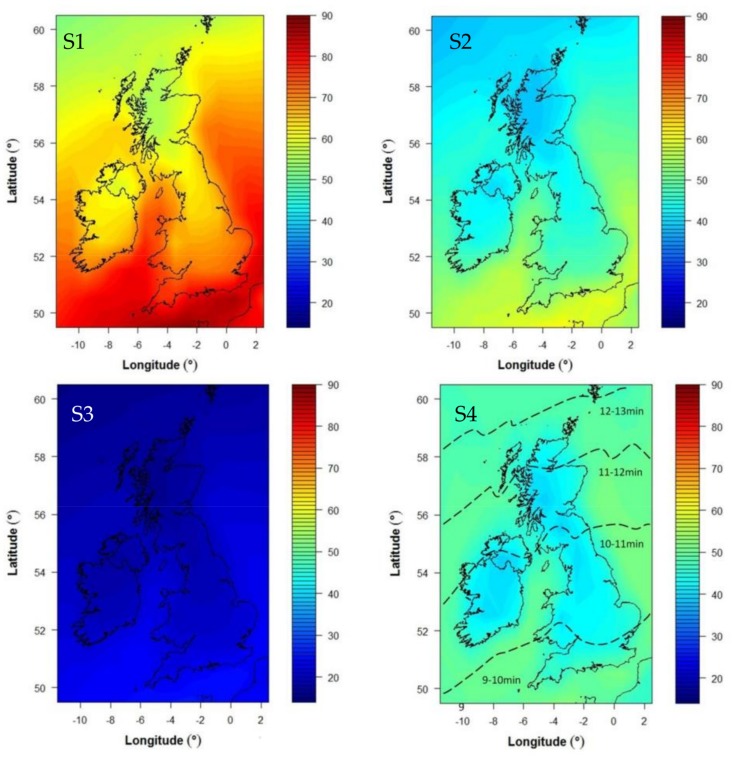
Calculated UVR doses in SEDs achievable from March-September on a randomly oriented vertical surface based on a 10-year UVR climatology [18] and daily exposures of <1 SED around noon for scenarios S1–S4 (Table 2). S1–S3 show the dose achieved after daily 9 min exposures at noon with 35% of skin area exposed March–September (S1). 10% of skin area in March–May and September, 35% June–August (S2). 10% of skin area throughout March–September (S3). S4 indicates the dose acquired after an exposure for a time equivalent to approximately 1 SED on 35% of skin area between June–August (no exposure in other months). For S4, exposure bands are labelled in minute intervals with the time to achieve 1 SED. While incident solar radiation is independent of skin area exposed, it is only effective for vitamin D synthesis when it falls on exposed skin. Therefore, the incident UVR has been scaled by the skin area for S2 and S3 to give “effective UVR” relative to the baseline skin area (35%) exposed in S1, which has a scaling factor of 1. See Method Section 2.3 for further details. The vertical color gradient key shows the number of indicative SED such that pale blue reflects achievement of the target dose (≥38 SED; S2 and S4), dark blue shows failure to achieve target (S3), and yellow-red shows the target is more than achieved (S1). The data illustrated are equivalent to E in Figure 1 for scenarios S1–S4 while the target dose (38 SED) is C in Table 3.

The values (ABCD) identified in Figure 1 are shown in Table 3. Figure 2 shows the baseline calculation of achievable SED across the UK for 35% of skin area exposed for 9 min daily from March–September, which was adjusted to a vertical surface (E in Figure 1) for comparison with the target summer dose required (38 SED: C in Figure 1, see Table 3). The other panels of Figure 2 show the equivalent calculation for the remaining scenarios in Table 2.

Exposure on all days is assumed, whatever the weather, and that, when the sun is shining, shade is not sought. Clouds have been accounted for in the underlying climatology and, on days with broken clouds or thin clouds, a significant UVR dose can still be received. However, acknowledging that rain may discourage exposure, we note that, when clouds are thick enough to produce persistent rain, UVR exposure is reduced. However, such occasions contribute a small amount to the total seasonal UVR. Therefore, avoiding exposure on days with prolonged rainfall during lunchtime would not be overly detrimental to vitamin D status. Further advice on handling days with no exposure is given in the discussion (Table 4). Extremes of summer weather and alternative behaviors are not addressed.

The largest assumption in the irradiance calculations is that of an uninterrupted sky view (e.g., standing in an open field with no horizon obstructions). Low horizon obstructions that are not casting a direct shadow on a person introduce little uncertainty, but, for city dwellers, the recommended exposure times may be insufficient in “city canyons” [26] and more open spaces like plazas or parks are preferred sites for gaining UVR exposure.

## 4. Discussion

The study results are theoretical even though they are based on real-life exposures and full, all-weather climatology. They illustrate what would be possible if the exposure regime used in the modeling were followed, recognizing that not everyone currently subscribes to such practices of either dress or sun exposure. The product of the skin area exposed and exposure time are theoretically adjustable provided that their product remains the same (and exposure is during the hour either side of solar noon). Thus exposure time could be reduced if the skin area exposed were increased. Reducing skin area and increasing exposure time is not recommended since this incurs the risk of erythema. Exposure for 9 min at other times of day will not be as effective for vitamin D synthesis and cannot fully substitute for exposure during the lunchtime period.

The results show that, for the white Caucasian population, under most of our scenarios, vitamin D needs can be met by regular, short, noontime exposures (see Figure 2). A short exposure (resulting in a dose <1 SED) is always less than 15 min even in Scotland (scenario S4). Provided that, at least during June–August, about 35% of skin surface area is exposed such as when wearing a T-shirt and shorts or a skirt, exposure time can be as little as nine minutes (scenarios S1 and S2). Therefore, requirements can be met through low level UK sunlight exposure and without recourse toward oral vitamin D supplements. Only scenario S3 (just hands and face, i.e., ~10% skin surface exposed throughout six months of the warmer seasons) failed to provide sufficient vitamin D synthesis. In contrast, scenario S1 sun-exposure practice (35% surface exposed throughout six months of warmer seasons) provided more exposure than needed while being less feasible for temperature reasons.

The fact that sunlight exposure is an important source of vitamin D has long been assumed [7,8], but it is usually expressed as casual exposure or “short periods outdoors” [27] without being fully quantified or in relation to specific situations and other metrics e.g., the personal minimal erythema dose (MED) [28] with the uncertainties that it brings [29]. The strength of this work lies in its novel use of UK datasets [11,17] directly linking human in vivo exposure to sunlight and vitamin D outcomes along with a detailed assessment of the UVR climate. Two major determinants of cutaneous vitamin D synthesis, which include the weather and human characteristics and behaviour, have multiple potential combinations. Both have been represented by clearly specified situations with the weather represented by 10-year all-weather climatology and the human aspect represented by the results of previous substantial direct in vivo research and then extended within defined exposure scenarios. Note that exposures at alternative times of day would have to be longer than nine minutes and would incur a lower benefit:risk profile due to the lower proportion of UVB available in the incident UVR. Results, therefore, represent a typical outcome for an average year of weather and for a “normal” person (97.5% of population) following the specified exposure pattern. As such, they provide examples of the sun exposure possibilities for vitamin D synthesis and quantify these in a simple way suitable for public health guidance. Table 4 shows how the results of Scenario 2 can be presented for the public and health professionals. Of the four scenarios, S3 is ineffective and exposing only the hands and face throughout the summer will not meet vitamin D requirements. The remaining three scenarios provide viable exposure options but S4 is location dependent and, therefore, does not allow for a simple country-wide message while the temperature would likely dissuade from exposing 35% of skin area in the colder months (S1). Therefore, S2 is the best scenario for a country-wide public health message.

It is important to recognize that exposed skin should be unprotected as specified in Table 4 and that protection includes cosmetics and moisturizers with many containing a sun-protective element. If the face is protected in this way, then the guidance in Table 4 can still be followed by exposing alternative skin area to compensate for the protected facial region. For example, the upper chest (equivalent to wearing a V-neck shirt), or an area of the upper arm.

There is public health guidance on minimizing sun exposure and the damaging effects of elongated sun exposure particularly in the noon hours, which might appear to contradict our work. However, in quantifying the UVR exposure required to meet vitamin D needs, we have used sunburn, i.e., visible reddening of the skin following sun-exposure, as a proxy for skin cancer risk in line with the approach of sun protection campaigns. It is seen that UVR can cause skin cell DNA damage even at sub-erythemal doses in white-skinned people [30,31] and, if incompletely repaired, this could potentially lead to mutagenesis. Therefore, there may still be some skin cancer risk even at low UVR doses. Currently, however, sunburn is a frequent phenomenon and it is evident that over-exposure to solar UVR is common [32]. The data we provide removes any justification for such over-exposure by demonstrating that low dose UVR is adequate for gaining vitamin D.

The subject of sun exposure and its influence on our health has become controversial and confusing to the public. The prevailing public health message has been that sun exposure is detrimental, which presents a major health burden in white-skinned populations. However, sun exposure is demonstrably the major source of vitamin D and required for musculoskeletal health. The relative benefit and risk become a matter of dose, but prior to the work presented in this manuscript, UVR doses for vitamin D across the UK had not been quantified. Now it is possible to provide advice and choice to the public in maintaining adequate vitamin D status, i.e., through the utilization of the sunlight source and/or enhanced oral intake. The choice may be further influenced by medical, social, and cultural factors that limit skin exposure such as in individuals who practice photoprotection and sun-avoidance, i.e., those prone to skin cancer, suffering from photosensitivity conditions, or who traditionally wear more covering clothing. It should be recognized that UVR can also have deleterious effects on health of the eye if not adequately protected and, conversely, UVR exposure of the skin has been shown experimentally to have potentially beneficial effects other than vitamin D synthesis [4]. This includes nitric oxide release, which may lower blood pressure and protect against cardiac disease and induction of antimicrobial proteins. These are areas of ongoing research [4].

In applying this work at the community level, it should be noted the model is based on previous results involving healthy adult volunteers (male and female, age 20–60 years). The simulated summer sun exposures have not been conducted in other age groups. Observational studies of sunlight exposures in teenagers are generally consistent with the results of the adult cohorts, which indicates pre-vitamin D synthesis is not markedly different [2]. It has long been supposed that the older-aged are disadvantaged in terms of skin synthesis of vitamin D because they have lower levels of precursor 7DHC [33]. Until their relative responses are quantified, care should be taken in applying the results to an older population. The advice does not apply to darker skinned populations (skin type V and VI) but similar work based on skin type V in vivo studies shows that the equivalent nine-minute exposure time for white Caucasians is estimated to be 25 min for skin type V [34]. As recent NICE guidance [35] has identified, tailored messages to specific groups are required based on benefits and risks of sunlight exposure.

Extrapolating this work to locations outside the UK would require assessment of the local UVR climatology, which is now available for Europe [36] and then its use to assess first a “safe” exposure (time for <1 SED at summer solstice noon) and then the length of the vitamin D winter [36]. Both of these variables are controlled predominantly by latitude. To the south of UK latitudes (which cover 50–60° N) 1 SED will be reached at increasingly shorter times when the latitude decreases while the vitamin D winter becomes shorter and there is a need for fewer stores to last through the shorter winter period, which theoretically allows exposure times to be decreased even further. To the north of UK latitudes, the time to reach 1 SED increases and so does the length of the vitamin D winter. It becomes increasingly difficult to acquire sufficient vitamin D through practical sun exposure alone. Nonetheless, our methods and the vitamin D production rates for white Caucasians can be applied at any latitude to determine the feasibility of maintaining vitamin D status through sun exposure.

## 5. Conclusions

We have exemplified a sun exposure regime that meets vitamin D requirements for white Caucasians. This is practical to achieve in everyday life, but provides only a fraction of a sunburn dose for a healthy adult even at the height of summer. This pivotally enables choice in obtaining vitamin D through appropriate short sunlight exposures or through oral vitamin D intake as defined by the UK nutrition agency (SACN [3]). White-skinned people in the UK (and similar latitudes) are able to meet vitamin D requirements (defined as remaining at or above 25 nmol/L 25(OH)D throughout winter) by spending nine minutes outdoors at lunchtime from March to September or for nine to 13 min, dependent on South-North geographical location, June–August, in season-appropriate clothing. Where such sun exposure is impractical or not desired, dietary sources of vitamin D (food, food fortification (country dependent), and vitamin supplements) should be assessed to ensure an adequate supply of the vitamin even though sun exposure is still likely to make some seasonal contribution to vitamin D status.

## Figures and Tables

**Table 1:** The previous human in vivo studies that were incorporated into the calculation of required UK summer sunlight exposure and the information they provided.

Study	Study Type Volunteers	Relevant Measures	Input
Webb et al. 2010 [11]	Observation Adult white Caucasian (*n* = 109; median age 44 (range 20–60) years; 78%F, 22%M)	Monthly 25(OH)D	September maximum 25(OH)D February minimum 25(OH)D Winter rate of 25(OH)D spend
Rhodes et al. 2010 [17]	Intervention Adult white Caucasian (*n* = 109; median age 35 (range 20–60) years; 68%F, 32%M)	25(OH)D response to 6 weeks of regular simulated sun	Increase in 25(OH)D for unit exposure to sunlight (skin types I–IV *)
Kazantzidis et al. 2015 [18]	Climate modelling n/a	Detailed UVR climatology of UK, validated against ground based measurements	Available UVR on 1° latitude by 1° longitude grid includes altitude and all weather 2003–2012

* Sun-reactive skin types I–VI were defined by Fitzpatrick [20]. These are based on sunburn and suntan ability and physical characteristics.

**Table 2:** The four noontime UVR sunlight exposure scenarios.

Scenario	Skin Area	Months	Time
S1	35% (face, hands, forearms, lower legs)	March–September	Constant
S2	35% (face, hands, forearms, lower legs)	June–August	Constant
	10% (face and hands)	March–May, September	
S3	10% (face and hands)	March–September	Constant
S4	35% (face, hands, forearms, lower legs)	June–August	Varies with latitude (equivalent to 1 SED) *

* The time to reach 1 SED was calculated as a function of latitude and, subsequently, used as latitude-dependent exposure time rather than using a single exposure time for the whole country, which was done in other scenarios.

**Table 3:** The intermediate results (A–D from Figure 1) and final outcome of each scenario. E > C indicates that, by following the exposure scenario, a sufficient vitamin D level can be synthesized in the skin to maintain winter vitamin D status with 25(OH)D ≥ 25 nmol/L for 97.5% of the population.

Method Step	Result
End summer month	September
End summer 25OHD target, A ^+^ (nmol/L)	80.5
Monthly 25OHD spend, B (nmol/L/month)	6.25
Summer dose required, C (SED)	38 *
Acceptable daily dose (SED)	1
Time for fixed daily dose (S1–3), D (minutes)	9
Time range (S4) for daily dose of 1 SED at noon in June. Time (minutes) varies with latitude from S England to N Scotland	9–13
S1: E > C 35% skin area March–September	Y
S2: E > C 10% skin area March–May + September plus 35% skin area June–August	Y **
S3: E > C 10% skin area all summer	N
S4: E > C 35% skin area, June–August, D adjusted for latitude to give 1 SED	Y

^+^ Ensures 97.5% population remain ≥ 25 nmol/L in February and 50% will be ≥ 50 nmol/L [8]. * The dose is calculated using a horizontal surface. The adjustment for a vertical body has been made in calculation of the exposure received at the skin under a range of scenarios [23] (see Section 2.5). ** Easily achieved in southern England and marginal in northern Scotland.

**Table 4:** Summary for practical application of results (Scenario 2).

Exposures are assumed to occur during normal lunchtime hours (approximately 12–2 pm during British Summer Time, which is one hour either side of the solar noon).
Exposure should occur every day * during the months from March to September. If a day is missed, double exposure time should not be pursued the next day. If there is a wish to compensate, more skin area could be exposed but for the same short time.
Exposure should be in an open place if possible and in direct sun when available (i.e., without seeking shade for this short period).
Exposed skin should be unprotected (no sunscreen, make up, or clothing e.g., tights)
During the months June–August, about 1/3 of skin area should be exposed. This is equivalent to face, hands, forearms, and lower legs, but areas are interchangeable so if the face is protected then upper arms or upper chest might be exposed instead.
During the remaining cooler months, only hands and face (or equivalent) need to be exposed although larger areas would be an advantage when appropriate.
***White-skinned people need a daily 9 min exposure*.**

* The calculations account for an all-weather climatology but little UV exposure will be gained during periods of heavy rain due to cloud cover. There is no need to get wet. Take exposure for the day when it is not raining or simply miss out on a very wet day.

## Supplementary Materials

The following are available online at http://www.mdpi.com/2072-6643/10/4/497/s1. File Webb_S1.docx containing details of all underlying data.
