# Supplementary material for: Meeting Vitamin D Requirements in White Caucasians at UK Latitudes: Providing a Choice
Source: Nutrients. 2018 Apr 17;10(4):497. doi: 10.3390/nu10040497 (PMC5946282; doi:10.3390/nu10040497)
Supplement: Supplementary file 1 [file nutrients-10-00497-s001.pdf]

Webb et al. Supplementary Information

Data Availability:

The underlying modelled climatology data and their validation against ground-based data have been previously published [reference 15]. The model, all model input data, and the data against which the model was validated are freely available online from international geophysical data centres.

The model itself is available at http://www.libradtran.org

The input data are available from:

https://ozoneaq.gsfc.nasa.gov/data/toms/ (TOMS ozone)

https://disc.sci.gsfc.nasa.gov/Aura/data-holdings/OMI (OMI ozone)

http://modis.gsfc.nasa.gov (Cloud cover and optical depth; Aerosol optical depth)

http://www.temis.nl/data/topo/dem2grid.html (Digital elevation data)

The measured ground-based data used for validation are available at http://www.woudc.org

The full *in vivo* human data set is available from Professor L.E. Rhodes.

The data for circulating 25OHD by month in a white Caucasian adult population was previously published [reference 8, Figure 1].

The change in circulating 25OHD after a 6 week course of simulated sunlight exposures has been previously published [reference 14, Figure 1].

Raw data used for regression of February on September 25OHD levels for healthy white Caucasian adults is given below.

id	diagnosis	photosen	sep25ohd	feb25ohd
121	Healthy	0	34.2	21.8
122	Healthy	0	19.9	14.1
123	Healthy	0	19.4	21
124	Healthy	0	16.8	7.5
125	Healthy	0	52.9	38.3
126	Healthy	0	13.7	8.9
127	Healthy	0	28.7	16.2
128	Healthy	0	22	13.1
129	Healthy	0	25.5	11.2
130	Healthy	0	10.4	3.9
131	Healthy	0	17.9	11.4
132	Healthy	0	30.3	16.2

133	Healthy	0	31.7	18.2
134	Healthy	0	28.7	19.5
135	Healthy	0	15.8	6.1
136	Healthy	0	35.7	10.4
137	Healthy	0	8.1	4.1
138	Healthy	0	22.7	22
139	Healthy	0	41.7	35.7
140	Healthy	0	28.4	13.7
141	Healthy	0	27.7	30.4
142	Healthy	0	31.2	20.4
143	Healthy	0	17.7	15.7
145	Healthy	0	31.4	14.1
146	Healthy	0	29.1	19.6
147	Healthy	0	24.6	22.8
148	Healthy	0	25.7	21.2
150	Healthy	0	29.3	18.2
151	Healthy	0	17.4	7.5
152	Healthy	0	39.3	24.5
154	Healthy	0	23.5	20
155	Healthy	0	28.7	16.8
156	Healthy	0		22.9
157	Healthy	0	28	12.3
159	Healthy	0	45.6	42.6
160	Healthy	0	33.9	20.9
161	Healthy	0	22.5	9.5
162	Healthy	0	23	16.9
163	Healthy	0	51.4	42.9
164	Healthy	0	16.7	8.3
165	Healthy	0	11.7	4.4
166	Healthy	0	9.9	4.4
168	Healthy	0	38.9	16.9
169	Healthy	0	29.5	26.5
170	Healthy	0	23.4	10.9
171	Healthy	0	27.9	17.3
172	Healthy	0	5.4	
176	Healthy	0	29.9	14.1
178	Healthy	0	29.8	14.8
179	Healthy	0	30.3	33
181	Healthy	0	38.4	22.4
182	Healthy	0	19.2	15.5
183	Healthy	0	30	20.3
184	Healthy	0	12.7	5.7
185	Healthy	0	19.6	4.7
186	Healthy	0	22.4	11.8

187	Healthy	0		
189	Healthy	0	39.7	18.2
190	Healthy	0	29.5	15.8
191	Healthy	0	18.7	14.6
192	Healthy	0	38.7	25.3
193	Healthy	0	35.1	20.9
194	Healthy	0	19.4	13.7
195	Healthy	0	36.3	21.1
196	Healthy	0	74.7	46.2
197	Healthy	0	32.9	14.6
198	Healthy	0	29.2	11.4
199	Healthy	0	29.2	21.1
200	Healthy	0	23.8	23.8
201	Healthy	0	26	11.9
202	Healthy	0	12.1	11
203	Healthy	0	35.5	15.3
204	Healthy	0	32.4	23.4
205	Healthy	0	47.6	31.3
206	Healthy	0	13.9	8.1
207	Healthy	0	40.9	28
208	Healthy	0	26.5	24.2
209	Healthy	0	34.4	21.7
210	Healthy	0	23.1	18.2
212	Healthy	0	35	30.3
213	Healthy	0	19	37.4
214	Healthy	0	24.8	19.4
215	Healthy	0	54.3	34.7
216	Healthy	0	39.7	22.8
217	Healthy	0	30.5	13.1
219	Healthy	0	18.5	19.3
220	Healthy	0	31.3	22.4
221	Healthy	0	28.2	15.1
222	Healthy	0	24.2	20.3
223	Healthy	0	46.3	25.9
224	Healthy	0	32.3	8.6
226	Healthy	0	31	22.8
227	Healthy	0	35.3	18.9
228	Healthy	0	31.3	21
229	Healthy	0	49.1	17.8
230	Healthy	0	27.5	19.5
232	Healthy	0	14.7	6.9
233	Healthy	0	22.4	23.9
234	Healthy	0	25.3	15.1
235	Healthy	0	39	26.6

237	Healthy	0	21	10
238	Healthy	0	30.4	10.3
239	Healthy	0	28.5	13.2
240	Healthy	0	17.6	7.2
241	Healthy	0	31.9	26.6
242	Healthy	0	20.3	9.6
243	Healthy	0	31.6	27.8
244	Healthy	0	38.1	20.6
245	Healthy	0	30.6	12.3
